# Neural mechanisms for emotional contagion and spontaneous mimicry of live facial expressions

**DOI:** 10.1098/rstb.2021.0472

**Published:** 2023-04-24

**Authors:** Joy Hirsch, Xian Zhang, J. Adam Noah, Aishwarya Bhattacharya

**Affiliations:** ^1^ Brain Function Laboratory, Department of Psychiatry, Yale School of Medicine, New Haven, CT 06511, USA; ^2^ Department of Neuroscience, Yale School of Medicine, New Haven, CT 06511, USA; ^3^ Department of Comparative Medicine, Yale School of Medicine, New Haven, CT 06511, USA; ^4^ Wu Tsai Institute, Yale University, PO Box 208091, New Haven, CT 06520, USA; ^5^ Haskins Laboratories, 300 George Street, New Haven, CT 06511, USA; ^6^ Department of Medical Physics and Biomedical Engineering, University College London, London WC1E 6BT, UK; ^7^ Yale College at Yale University, New Haven, CT 06511, USA

**Keywords:** interactive face-processing, functional near-infrared spectroscopy (fNIRS), hyperscanning, facial mimicry, emotional contagion

## Abstract

Viewing a live facial expression typically elicits a similar expression by the observer (facial mimicry) that is associated with a concordant emotional experience (emotional contagion). The model of embodied emotion proposes that emotional contagion and facial mimicry are functionally linked although the neural underpinnings are not known. To address this knowledge gap, we employed a live two-person paradigm (*n* = 20 dyads) using functional near-infrared spectroscopy during live emotive face-processing while also measuring eye-tracking, facial classifications and ratings of emotion. One dyadic partner, ‘Movie Watcher’, was instructed to emote natural facial expressions while viewing evocative short movie clips. The other dyadic partner, ‘Face Watcher’, viewed the Movie Watcher's face. Task and rest blocks were implemented by timed epochs of clear and opaque glass that separated partners. Dyadic roles were alternated during the experiment. Mean cross-partner correlations of facial expressions (*r* = 0.36 ± 0.11 s.e.m.) and mean cross-partner affect ratings (*r* = 0.67 ± 0.04) were consistent with facial mimicry and emotional contagion, respectively. Neural correlates of emotional contagion based on covariates of partner affect ratings included angular and supramarginal gyri, whereas neural correlates of the live facial action units included motor cortex and ventral face-processing areas. Findings suggest distinct neural components for facial mimicry and emotional contagion.

This article is part of a discussion meeting issue ‘Face2face: advancing the science of social interaction’.

## 1. Introduction

It has long been recognized that live dyadic interactions frequently include unconscious imitation (mimicry) during reciprocal interactions [1–3]. For example, it is also commonly observed in conversations between dyads, where copying non-verbal and verbal features is observed in addition to the explicit content of the speech [4–6]. Although the social function of these unconscious ‘imitation’ behaviours is not well understood, it has been proposed that facial mimicry and other forms of converging interactive behaviours represent prosocial responses that generally increase social affiliation [7]. For this reason, dyadic mimicry has been referred to as ‘social glue’ [8] and represents a high-priority behavioural topic for investigation of live, spontaneous, social interactions.

The theory of embodied emotion proposes a relationship between the neural systems that underlie dynamic facial mimicry and the processing of emotion [9–12]. Consistent with this theory, it has been noted that simulation of a perceived facial expression partially activates the corresponding emotional state, providing a basis for inferring the underlying emotion of the expresser [1]. The significance of this question lies with the hypothesis that facial mimicry may represent a native biological mechanism that supports the conveyance of emotion between interacting humans and serves as a mechanism that underlies interpretation of facial expressions. Behavioural evidence for such a relationship between facial mimicry and emotional processing has been provided by a paradigm where mimicry of facial expressions was blocked by using a face stabilizer consisting of a pencil in the mouth of the ‘Face Watcher’. Findings confirmed that prevention of the physical mimicry of the facial expression impaired ability to recognize emotions [13]. A similar experiment that blocked facial mimicry was also performed while recording electroencephalogram (EEG) signals during passive viewing of facial expressions with emotional content including anger, fear and happiness. Mu desynchronization was observed when participants could freely move their facial muscles but not when their facial movements were inhibited. The findings were interpreted as consistent with a neural link between motor activity, automatic mimicry of facial expressions and the communication of emotion [14].

This hypothesized neural link between the encoding of facial mimicry and neural activity within the emotion-processing systems has also been investigated using botulinum toxin to immobilize frown muscles while neuroimaging using fMRI. Self-initiated frown expressions during the effective period of the toxin resulted in reduced responses in the amygdala (a brain region known to be sensitive to emotional stimuli) during gaze at angry faces relative to the pre-botulinum administration. Results were interpreted as support for the hypothesis that mimicry of passively viewed emotional expressions provides a physiological basis for the social transfer of emotion [15]. However, the neural basis for this relationship remains an active and high-priority question.

Humans are thought to be profoundly social [16], and the dynamic and expressive human face is a universally recognized social ‘meter’ [17]. Skill in accurately translating facial expressions indicating another person's situation or emotional status is an everyday requirement for successful social relationships and conventional interpersonal encounters [18,19]. The focus on faces and eyes [20–22] has long provided an entry point to investigate the neural processing of salient visual features and models of face-processing [23–27]. Neural processing of social, cognitive and emotional behaviours is also embedded in decades of neuroscience based on behavioural, electrophysiological, computational and functional imaging contributing to an ever-expanding knowledge base of the social brain [16,28–30]. The theoretical frameworks for face and social processing merge with increasing focus on natural and spontaneous interactions between individuals.

Current understanding of face and social processing is primarily based on static representations of each. However, models of face-processing, eye contact and social mechanisms including verbal interactions come together with investigations of live and dynamic facial expressions [31–34], spoken language [35,36] and the related dyadic sharing of emotional information. Here we employ live and spontaneous emotion-expressing faces as primary social stimuli. Measures of facial classifications and associated neural responses based on functional near-infrared spectroscopy, fNIRS, and behavioural ratings are applied to investigate cross-brain (dyadic) effects of emotional faces.

The organizational principles of neural coding underlying dynamic and interpersonal social interactions are critically understudied relative to their importance for understanding basic human behaviours in both typical individuals and psychiatric, neurological, and/or developmental disorders [37–43]. This barrier to progress is largely due to technical limitations related to the need to acquire neuroimaging data on two or more individuals simultaneously while engaging in interactive behaviours. Conventional neuroimaging methods, functional magnetic resonance imaging (fMRI) and positron emission tomography (PET), are generally constrained to single-subject studies that do not include ecologically valid interactions. Here we address this knowledge gap using a two-person paradigm and neuroimaging technology specialized for hyperscanning in natural conditions. This novel and emerging focus on the dyad rather than single brains interweaves and extends models of dynamic face-processing and interactive social, cognitive and emotional neuroscience

**Figure 1.**
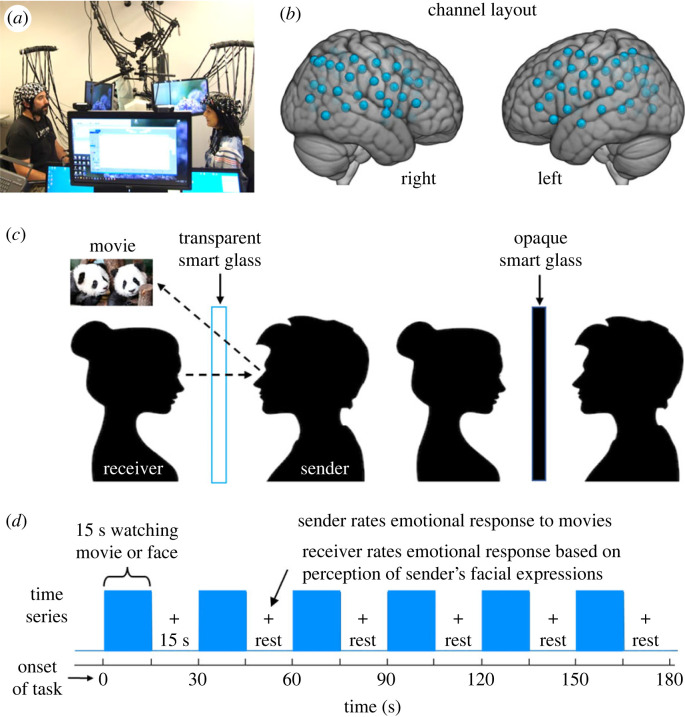
(*a*) Set-up for simultaneous neuroimaging of interacting participants separated by a glass panel, i.e. ‘smart glass’ (picture permissions obtained). (*b*) Channel layout. Right and left hemispheres of a single-rendered brain illustrate median channel locations (blue dots) for 58 channels per participant. Montreal Neurological Institute (MNI) coordinates for each recording channel and corresponding anatomical locations were determined with NIRS-SPM [51]. (*c*) Paradigm schematic. The ‘smart glass’ divider is transparent during the task (blue bars in (*d*)) and opaque during rest periods (15 s blank in (*d*)). (*d*) Time series for a single run.

Obstacles related to imaging neural activity acquired from two interacting individuals are largely addressed using the emerging neural imaging technology fNIRS, which acquires blood oxygen level-dependent (BOLD)-like signals using optical techniques rather than magnetic resonance [44]. This enables functional brain imaging in natural (upright and face-to-face) conditions during hyperscanning of two interacting individuals [45,46]. The signal is based on differential absorption of light by oxyhaemoglobin (OxyHb) and deoxyhaemoglobin (deOxyHb), a proxy for neural activity [47–49]. Although fNIRS has been widely applied for neuroimaging of infants and children, the technology has not been widely applied to adult cognitive research, largely owing to sparse optode coverage and low spatial resolution (approximately 3 cm) relative to fMRI. However, tolerance to movement and the absence of conditions such as a high magnetic field, constraining physical conditions, the supine position and loud noise recommend this alternative technology for two-person live interactive studies. Signals are acquired at roughly every 30 ms, which provides a signal-processing advantage for measures of functional connectivity [50] and neural coupling [32,45] even though the signal is the (slow) haemodynamic response function. Additional technical advances optimize two-person neuroimaging, including extensive bilateral optode coverage for both participants, ‘smart glass’ technology to occlude or reveal views according to a block paradigm that creates ‘rest’ blocks in the time series (figure 1*a*), and the multi-modal acquisitions including simultaneous facial classifications, eye-tracking, behavioural ratings and live interactive paradigms.

Topographical maps of retinal space are distributed throughout the cortex and provide a framework for cortical organization of information-processing [52]. Face-processing modules have been proposed within this framework and are supported by evidence of hierarchical specializations for faces along the ventral stream [19,25]. These prior foundational findings predict that live and interactive face-processing may indeed engage these and additional neural systems above and beyond those that have been so thoroughly studied using conventional single-subject, static and simulated face methods. Additional mechanisms for live and interactive faces are also predicted by the interactive brain hypothesis [53], which proposes specialized neural and cognitive effects due to live interactions between individuals. Specifically, we have shown that live and interactive face-processing mechanisms intersect known social mechanisms and engage right processes including the right angular gyrus, and dorsal stream regions including somatosensory association cortex and supramarginal gyrus [32–34]. We hypothesize that these dorsal regions will also underlie encoding of emotional information shared during spontaneous dyadic face interactions.

Spontaneous facial mimicry has been widely investigated for its putative role in communication and affiliation. It has been suggested that this mechanism is implemented by prefrontal activity that mediates top-down influences on sensory and social systems [54,55]. This suggestion is also consistent with fMRI findings where participants observed an emotional facial expression and were requested to emit an inconsistent expression. Findings of this ‘Stroop-like’ task revealed activity in right frontal regions previously found active during the resolution of sensory conflict [56], activity in supplementary motor cortex as expected with engagement of facial muscles employed in mimicry, and activity in posterior superior temporal sulcus as predicted based on known social systems [28]. Findings were interpreted as evidence for a frontal and posterior neural substrate associated with dynamic and adaptive interpretation and encoding of intentional facial expressions [57]. fMRI findings have also suggested a central role for right inferior frontal gyrus in a task where emotional expressions were intentionally simulated on command [58]. However, the neural correlates of facial mimicry have not been directly investigated in a live two-person paradigm without the confound of cognitive tasks expected to engage associated executive and conflict resolution functions. The significance of these questions and mechanisms is enhanced by prior observations of impairments in automatic mimicry in children with autism spectrum disorder, ASD, referred to as the ‘broken mirror’ theory of autism [59].

Here we focus on a novel multi-modal approach using live dyadic interactions, facial classifications, eye-tracking, neuroimaging using fNIRS, and subjective behavioural reports of conveyed affect during passive gaze at an emotive face. We aim to isolate neural correlates that underlie spontaneous mimicry and emotional contagion. The large body of behavioural evidence suggests that emotional contagion via passive viewing of a facial expression is linked to spontaneous mimicry of the expression. These observations suggest that the neural systems for emotional contagion and facial mimicry may be shared. However, it is not known if these neural systems are separate components of an integrated biological complex or if these systems are bundled together within interactive face-processing and social systems. Here we investigate these alternatives. *A priori* temporal–parietal expectations include right hemisphere social systems such as regions within the right junction [28], systems associated with ventral stream face-processing including the lateral occipital cortex and superior temporal gyrus [19,52], and regions previously associated with live and interactive faces including right dorsal and temporal-parietal regions [34], in addition to motor cortex associated with faces.

## 2. Methods

### (a) Participants

Adults 18 years of age and older who were healthy and had no known neurological disorders (by self-report) were eligible to participate. The study sample included 40 participants (26 women, 12 men and 2 identified as another gender; mean age: 26.3 ± 10.5 years; 36 right-handed and 4 left-handed. Eleven participants identified as Asian, five as biracial, one as White Latin X, and 23 as White. Dyad types included eight female–female pairs, eight male–female pairs, two female–other pairs and two male–male pairs (see electronic supplementary material, tables S1 and S2). Counterbalancing for dyad type, gender and race was achieved by order of recruitment in a diverse population, which tended to distribute these potentially confounding factors, to avoid influence on findings. There were no repeated measures. The sample size was based on a power analysis and prior dyadic studies in real interactive conditions. It was determined that relative signal strengths (beta values) for task-based activation were 0.00055 ± 0.00103, giving a distance of 0.534. Therefore, 15 dyads (*n* = 30) were required to achieve a power of 0.80. Our sample size of 20 dyads (*n* = 40) provides greater than required confidence for our statistical analysis. Twenty dyads was our *a priori* targeted sample size. All participants provided written informed consent in accordance with guidelines approved by the Yale University Human Investigation Committee (HIC no. 1501015178) and were reimbursed for participation. Dyad members were unacquainted prior to the experiments and assigned in order of recruitment. Laboratory practices are mindful of goals to assure diversity, equity and inclusion, and accruals were monitored by regular evaluations based on expected distributions in the surrounding area. Each participant provided demographic and handedness information before the experiment.

### (b) Set-up

Dyads were seated 140 cm across a table from each other (figure 1*a*) and were fitted with an extended head-coverage fNIRS cap (figure 1*b*). As seen in figure 1*a,c*, separating the two participants was a custom-made controllable ‘smart glass’ window that could change between transparent and opaque states by application of a programmatically manipulated electrical current. Attached to the top and middle of the smart glass were two small 7-inch LCD monitors with a resolution of 1024 × 600 pixels. The monitors were placed in front of and above the heads of each participant, so the screens were clearly visible but did not obstruct their partner's face. Monitors displayed video clips (Movie Watcher only; figure 1*c*) and cued participants to rate subjective intensity and valence (positive or negative) of the affective experiences using the dial.

### (c) Paradigm

During the interaction, participants took turns in two aspects of a dyadic interactive task. One partner within a dyad, the Movie Watcher, watched short (3–5 s) video clips on the LCD screen (randomly presented using a custom Python script) while the other partner, the Face Watcher, observed the face of the Movie Watcher (figure 1*c*). Partners alternated roles as Movie Watcher and Face Watcher. Movies were presented in 3 min runs that alternated between 15 s of movies and 15 s of rest (figure 1*d*). There were five movies in each 15 s task block with the run. Each Movie Watcher saw a total of 60 movie clips (two runs) for each movie type, and there were three movie types, so each Movie Watcher saw a total of 180 movie clips. After each 15 s set of movie stimuli, Movie Watchers rated their affective responses with a dial on a Likert-type scale evaluating both valence and intensity (positive: 0 to +5, negative: 0 to −5) to the movie block. Face Watchers rated the intensity of their own affective feelings based on their experience of the Movie Watcher's facial expressions during the same period. The comparison of these affective ratings between dyads is reported to document the extent to which the emotion was communicated via a facial expression on an epoch-by-epoch basis. For purposes of description, ratings for all trials and all participants are represented graphically and summarized by a scatterplot that includes both within- and across-subject data. The affect ratings were also analysed on a dyad by dyad basis and the average correlations between all dyads are presented to represent the overall strength of the observed association (emotional contagion).

Facial expressions of both partners were acquired by cameras (recorded as part of the Python script) and analysed with OpenFace (details below). Each participant performed the Movie Watching and Face Watching tasks three times*,* including two runs for every movie type (‘adorables’, ‘creepies’ and ‘neutrals’, see below) for a total of six 3 min runs and a total duration of 18 min. Movie clips were not repeated. Similar to the affect of ratings above, facial action units (AUs) for all trials and all participants are represented graphically and summarized by a scatterplot that includes both within- and across-subject data. The mean intensity of the first principal component (PC) of facial AUs was also analysed on a dyad by dyad basis and the average correlation across dyads is presented to represent the overall strength of the observed association (facial mimicry).

### (d) Movie library of emotive stimuli to induce natural facial expressions

Emotionally evocative videos (movies) intended to elicit natural facial expressions were collected from publicly accessible sources and trimmed into 3–5 s clips. All video stimuli were tested and rated for emotive properties by laboratory members. The clips contained no political, violent or frightening content, and participants were given general examples of what they might see prior to the start of the experiment. The three categories of videos included: ‘neutrals’, featuring landscapes; ‘adorables’, featuring cute animal antics; and ‘creepies’, featuring spiders, worms and states of decay. Videos were rated prior to use in the experiment according to the intensity of emotions experienced (from 0 to 100 on a continuous-measure Likert-type scale; 0: the specific emotion was not experienced, and 100: emotion was present and highly intense) according to basic emotion types (joy, sadness, anger, disgust, surprise and fear). For example, a video clip of pandas rolling down a hill (from the 'adorables' category) might be rated an 80 for joy, 40 for surprise and 0 for sadness, fear, anger and disgust. Responses were collected and averaged for each video. The final calibrated set used in the experiment consisted of clips that best evoked intense affective reactions (except for the ‘neutrals’ category, from which the lowest-rated videos were chosen).

### (e) Instructions to participants

Participants were informed that the experiment aimed to understand live face-processing mechanisms and were instructed according to their role (i.e. Face Watcher or Movie Watcher). The Face Watcher was instructed to look naturally at the face of the Movie Watcher when the smart glass was clear. The Movie Watcher was instructed to look only at the movies and emote natural facial expressions during that same time period. Natural expressions (such as smiles, eye blinks and other natural non-verbal expressions) were expected owing to the emotive qualities of the movies. Participants were instructed not to talk during the runs, and eye-tracking in addition to the scene cameras confirmed compliance with directional gaze instructions.

### (f) Functional near-infrared spectroscopy signal acquisition and channel localization

Functional NIRS signal acquisition, optode localization and signal-processing, including global component removal, were similar to methods described previously [45,56,60–64] and are briefly summarized below. Haemodynamic signals were acquired using three wavelengths of light, and an 80-fibre multichannel, continuous-wave fNIRS system (LABNIRS, Shimadzu, Kyoto, Japan). Each participant was fitted with an optode cap with predefined channel distances. Three sizes of caps were used based on the circumference of the participants' heads (60 cm, 56.5 cm or 54.5 cm). Optode distances of 3 cm were designed for the 60 cm cap but were scaled equally to smaller caps. A lighted fibre-optic probe (Daiso, Hiroshima, Japan) was used to remove all hair from the optode holder before optode placement.

Optodes consisting of 40 emitters and 40 detectors were arranged in a custom matrix providing a total of 58 acquisition channels per participant. For consistency, the placement of the most anterior midline optode holder on the cap was centred one channel length above nasion. To ensure acceptable signal-to-noise ratios, intensity was measured for each channel before recording, and adjustments were made for each channel until all optodes were calibrated and able to sense known quantities of light from each laser wavelength [61,65,66]. Anatomical locations of optodes in relation to standard head landmarks were determined for each participant using a structure.io three-dimensional scanner (Occipital, Boulder, CO) and portions of code from the fieldtrip toolbox implemented in Matlab 2022a [67–71]. Optode locations were used to calculate positions of recording channels (figure 1*b*), and Montreal Neurological Institute (MNI) coordinates [72] for each channel were obtained with NIRS-SPM software [51] and WFU PickAtlas [73,74].

### (g) Eye-tracking

Two Tobii Pro x3–120 eye trackers (Tobii Pro, Stockholm, Sweden), one per participant, were used to acquire simultaneous eye-tracking data at a sampling rate of 120 Hz. Eye trackers were mounted on the screen facing each participant. Prior to the start of the experiment, a three-point calibration method was used to calibrate the eye tracker on each participant. The partner was instructed to stay still and look straight ahead while the participant was told to look first at the partner's right eye, then left eye, then the tip of the chin. Eye-tracking data were not acquired on a subset of participants owing to technical reasons associated with the loss of the signal for some participants for which the eye-tracking was not sensitive. The eye-tracking served to confirm that there was no eye contact between the Face Watcher and the Movie Watcher and, when not available, data from the scene cameras substituted for this confirmation. Thus, expected gaze directions were confirmed on all participants. This is important because it has been shown that mimicry is modulated by direct gaze [75–77].

### (h) Facial classification: validation of manual ratings and automated facial action units

Automated facial AUs were acquired simultaneously from both partners using OpenFace [78] and Logitech C920 face cameras to acquire facial features. OpenFace is one of several available platforms that provide algorithmically derived tracking of facial motion in both binary and continuous format. Automatic detection of facial AUs using these platforms has become a standard building block of facial expression analysis, where facial movements are described as dynamic conformational patterns of facial muscle anatomy. Although a direct relationship between distinct emotions and activation patterns has been postulated [79], here facial expressions are partitioned into discrete muscular components and dynamics without association with emotional labels. Participants rated overall affect intensity and valence rather than naming an emotion associated with the movie clip (Movie Watcher) or facial expression (Face Watcher). The facial AU analysis using OpenFace included 17 separate classifications of anatomical configurations.

Conventional methods to validate the relationship between emotions and facial expressions have employed manual codes [80,81]. For example, Ekman, Friesen & Hager developed a manual observer-based method for coding facial expression measurements [82] referred to as the facial action coding system (FACS). FACS provides a technique to record an objective description of facial expressions based on activations of facial muscles and has provided a foundation to link human emotions with specific human facial expressions. By contrast, application of the OpenFace platform in this investigation does not relate facial AUs to any specific emotion. Spontaneous expressions of the Movie Watcher are classified as discrete constellations of moving parts (AUs) and rated by the Face Watcher using a scale from −5 to +5 indicating affect valence and intensity. There is no inference with respect to a specific emotion.

**Figure 2.**
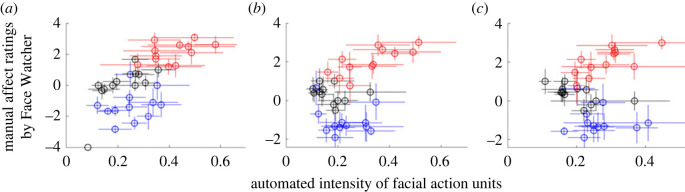
Manual affect ratings by face viewer averaged for all participants are plotted versus the averaged automated intensity measures of three representative AUs [78] from the Movie Watcher: Lip Corner Pull (12) (*a*), Lips Part (25) (*b*) and Chin Raiser (17) (*c*). Each dot represents a 15 s task block. The *y*-axis shows average affect rating of the Face Watcher based on the Movie Watcher's facial expression. The *x*-axis shows the AU intensity obtained by OpenFace for the 15 s block. The vertical error bars indicate the inter-rater reliability and the horizontal error bars indicate the variation in the facial stimuli presented to the Face Watcher. In spite of the highly variable facial expressions, Face Watchers consistently rated valence and intensity in accordance with expectations of movie types.

The application of spontaneous and live individual human facial expressions as a stimulus for the investigation of face-processing is novel. Dynamic faces constitute a high-dimensional stimulus space not previously explored by conventional experimental paradigms. To validate this dyadic methodology we plot the average manual affect ratings (*y*-axis) against average automatic measures of facial AUs (*x*-axis) for all three movie types: 'adorables' (red), 'neutrals' (black) and 'creepies' (blue), all 17 AUs, and all participants (see electronic supplementary material, figure S1*a*,*b*). Figure 2*a–c* (below) shows examples of three typical AUs: (*a*) 12 (Lip Corner Pull), (*b*) 25 (Lips Part) and (*c*) 17 (Chin Raiser). Each data point (circle) in the scatterplots represents averaged information from each 15 s task block (figure 1*d*). Vertical error bars indicate the s.e.m. of all ratings, which, on average, is ±0.34 s.e.m. across all blocks and AUs, and confirms a high level of consistency across participants who viewed the spontaneous and natural expressions from the many expressers (Movie Watchers). Horizontal error bars indicate the s.e.m. of AU intensities which, on average, is ±0.04 s.e.m. across all blocks and AUs. This relatively high variability of the facial AU intensities (*x*-axis) is consistent with a range of individual differences that consist of natural and spontaneous real human facial expressions from each of the individuals who participated in the experiment [83]. None of these individuals was a part of the research team and all were naive to the experimental details. These scatterplots and validity metrics illustrate that, in spite of the highly variable faces and facial expressions (*x*-axis), face raters (*y*-axis) consistently rated valence and intensity in accordance with movie types: ratings of the ‘adorable’ movies are in the positive range, ratings of the ‘neutral landscapes’ are in the ‘no-affect’ range and ratings of the ‘creepie’ movies are in the negative range. These inter-rater reliability scatterplots serve to validate this core variable of automated facial AUs acquired by OpenFace as a measure of live and spontaneous facial motion.

Given the uniform classifications of valence and intensity, a principal components analysis (PCA) was applied to represent the facial dynamics. The first principal component (PC1) was used as modulator of the neural data. This approach included all 17 facial AUs weighted according to their contribution. The Pearson's correlation coefficient of the PC1 AUs between the two partners was taken as an objective representation of facial movement mimicry from the Movie Watcher (expresser) to the Face Watcher (face viewer). The average of the correlation coefficients across all dyads is taken as the group measure of the strength of that association.

### (i) Functional near-infrared spectroscopy signal processing

Raw optical density variations were acquired at three wavelengths of light (780, 805 and 830 nm), which were translated into relative chromophore concentrations using a Beer–Lambert equation [84–86]. Signals were recorded at 30 Hz. Baseline drift was removed using wavelet detrending provided in NIRS-SPM [51]. In accordance with recommendations for best practices using fNIRS data [87], global components attributable to blood pressure and other systemic effects [88] were removed using a PCA spatial global mean filter [60,62,89] before general linear model (GLM) analysis. This study involves emotional expressions that originate from specific muscle movements of the face, which may cause artefactual noise in the OxyHb signal. To minimize this potential confound, we used the HbDiff signal, which combines the OxyHb and deOxyHb signals for all statistical analyses. However, following best practices [87], baseline activity measures of both OxyHb and deOxyHb signals are processed as a confirmatory measure. The HbDiff signal averages are taken as the input to the second level (group) analysis [90]. Comparisons between conditions were based on GLM procedures using NIRS-SPM [51]. Event epochs within the time series were convolved with the haemodynamic response function provided from SPM8 [91] and fitted to the signals, providing individual ‘beta values’ for each participant across conditions. Group results based on these beta values are rendered on a standard MNI brain template (TD-ICBM152 T1 MRI template [72]) in SPM8 using NIRS-SPM software with WFU PickAtlas [73,74].

### (j) General linear model analysis

The primary GLM analysis consists of fitting four model regressors (referred to as covariates) to the recorded data. For each 30 s block, there are 15 s of task, either movie viewing or face viewing (depending upon the condition), and 15 s of rest. During the 15 s task epochs, visual stimuli were presented to both participants: the Movie Watcher viewed movie clips on a small LCD monitor (figure 1*c*), and the smart glass was transparent so the Face Watcher could observe the face of the Movie Watcher. For each type of movie, the onsets and durations were used to construct the square wave block design model. The three movie types served as the first three covariates. The fourth model covariate (referred to as Intensity) was a modulated block design created to specifically interrogate the neural responses of the Face Watcher's brain by either the affective ratings or the facial AUs of the Movie Watcher.

## 3. Results

### (a) Affective ratings

**Figure 3.**
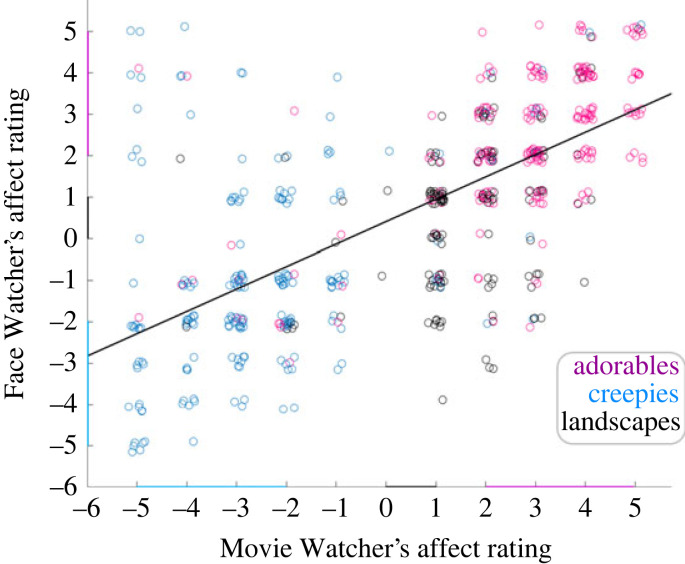
Scatterplot of the Movie Watchers' and Face Watchers' emotional affect based on ratings of valence and intensity. Red: 'adorables', blue: 'creepies' and black: 'landscape' movies. This scatterplot illustrates all within- and across-subject observations. For quantitative purposes, each dyad pair was analysed separately and the mean correlation across all dyads was *r* = 0.67 ± 0.04 s.e.m. The correlation between the two ratings is consistent with emotional contagion. That is, the expression on the face of the Movie Watcher (as rated by the Movie Watcher) tended to convey the emotion to the Face Watcher.

Average emotional ratings are represented on the scatterplot in figure 3, where the Movie Watchers' ratings of affect valence and intensity (*x*-axis) are plotted against the Face Watchers’ ratings (*y*-axis) for all three movie types: red, black and blue circles represent ‘adorables’, ‘neutrals’ and ‘creepies’, respectively. This graphical illustration confirms that ratings were generally matched within the dyad for both intensity and valence. In particular, expressions associated with the ‘adorable' movies (red) were ranked as higher than the ‘landscapes’ (black). The ‘creepy' movies tended to be more variable because a ‘cringe’ expression (negative valence) of the Movie Watcher, in some cases, elicited a jovial response (positive valence) from the Face Watcher. However, overall evidence for emotional contagion is provided by the average correlation (*r* = 0.67 ± 0.04 s.e.m.) between the ratings across all movie types.

### (b) Facial classifications

**Figure 4.**
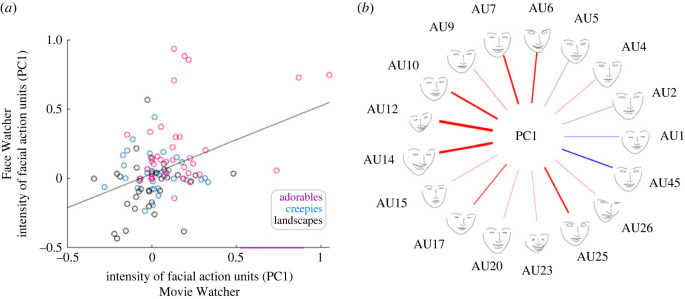
(*a*) Scatterplot of the Movie Watcher's and Face Watcher's PC facial AUs: Movie Watcher on the *x*-axis and Face Watcher on the *y*-axis. The scatterplot illustrates the relationship between the partners' facial AUs for each of the movie types (red, 'adorables'; blue, 'creepies'; and black, 'landscapes'). All 17 AUs are included in the dataset and are represented for each movie type and all participants. Quantification of the relationship between the expresser (Movie Watcher) and the responder (Face Watcher) is based on the average of the individual correlations for each dyad (*r* = 0.36 ± 0.11 s.e.m). Findings are consistent with the hypothesis of facial mimicry (sometimes referred to as sensorimotor simulation, [1]). That is, the expression on the face of the Movie Watcher showed some tendency to be automatically replicated on the face of the Face Watcher. (*b*) Illustration of the relative contributions of each AU to PC1. The AUs are shown on the outer circle by name. The thickness of each line represents the coefficient of the AU to PC1 and the colour of the line represents the valence: positive (red) or negative (blue) of the coefficient. PC1 explains 37 ± 1.6% s.e.m. of the total variance.

The facial AUs were acquired in real time simultaneously on both partners during the task (transparent glass) epochs. Evidence for facial mimicry is provided by the correlation between the first PC calculated for each participant and movie type and represented as shown in figure 4*a*. The *x-* and *y*-axes represent facial classifications based on the PC1 for each of the dyadic partners. The correlation (*r* = 0.36 ± 0.11 s.e.m.) between the two partners and across the movie types is taken as an objective representation of facial movement mimicry from the Movie Watcher to the Face Watcher. Together these findings are consistent with the hypothesis that facial mimicry is observed during the conveyance of affective perceptions [57,92,93].

Figure 4*b* illustrates the relative contributions (line thickness) of each of the facial AUs and their valence (red, positive; blue, negative) to PC1. As expected from prior reports, AU12 (zygomaticus major) and AU6 (orbicularis oculi) are relevant to positive facial reactions whereas AUs 4 and 5 associated with frowning are less relevant in this application [80,94]. Further descriptions of these specific AUs have been reported previously [78]. The PC1 accounted for 37 ± 1.6% s.e.m. of the total variance.

### (c) Neural responses: how does the expressive face of the Movie Watcher modulate the brain of the Face Watcher?

**Figure 5.**
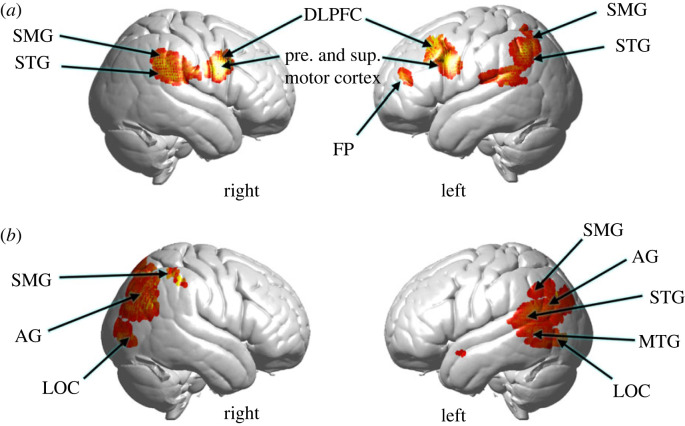
(*a*) The Movie Watcher's facial expressions as represented by the PC1 of facial AUs are applied as a modulator (covariate) of neural activity on the Face Watcher's fNIRS signals acquired while viewing the emotive face of the Movie Watcher (combined oxyhaemoglobin (OxyHb) and deoxyhaemoglobin (deOxyHb) signals). See electronic supplementary material, table S3 for cluster details. (*b*) The Movie Watcher's affect valence and intensity ratings applied as a modulator of neural activity of the Face Watcher's fNIRS signals acquired while viewing the emotive face of the Movie Watcher (combined OxyHb and deOxyHb signals). AG, angular gyrus; DLPFC, dorsolateral prefrontal cortex; FP, frontal pole; LOC, lateral occipital cortex; MTG, middle temporal gyrus; SMG, supramarginal gyrus; STG, superior temporal gyrus. See electronic supplementary material, table S4 for cluster details.

For each dyad, the principal component AU, PC1 AU, was applied to modulate the neural responses of the Face Watcher. Figure 5*a* shows increased activity in the right temporal–parietal junction, including the superior temporal gyrus, STG, and supramarginal gyrus, SMG, consistent with prior findings of interactive face gaze [32–34], and bilateral pre-and supplementary motor cortex, consistent with an additional motor response related to face-processing. See electronic supplementary material, table S3.

### (d) Neural responses: how does the affect rating of the Movie Watcher modulate the brain of the Face Watcher?

The average affect intensity and valence ratings for the Movie Watcher for each movie type were employed as a covariate on the neural responses of the Face Watcher using the GLM described above. Right temporal–-parietal junction regions (figure 5*b*) are observed adjacent and posterior to areas responsive to the facial AU shown in figure 5*a*, including SMG, AG and lateral occipital cortex, LOC. These regions have been implicated in facial and social processes including Theory of Mind [95]. Activity is also observed in left LOC and inferior, middle and superior temporal gyri in addition to the angular gyrus and supramarginal gyrus, suggesting a robust bilateral neural response during the conveyance of affect from the face of a dyadic partner. See electronic supplementary material, table S4.

## 4. Discussion

### (a) From the single brain to the dyad: a theoretical shift

Within a dyadic model, a single human brain is only one half of the fundamental social unit. The emerging development of two-brain functional imaging systems advances a paradigm shift from single brains to dyads. As a result, a new set of principles of dyadic functions that underlie the neural correlates for human cognition, perception and emotions come into focus, suggesting an important future direction. Although emotional contagion is recognized as a foundational feature of biological social interactions, the underlying mechanisms are not understood, partly owing to prior technical roadblocks to investigation of dyadic behaviours. It has been suggested that spontaneous facial mimicry may provide an interactive mechanism for the transfer of emotion from one person to another. As such, this putative mechanism is a focus for investigation of dyadic exchanges in this study.

We apply a dyadic neuroimaging approach enabled by optical imaging and multi-modal techniques to simultaneously investigate the mechanisms of facial mimicry and emotional contagion. One participant, the ‘Movie Watcher’, generated facial expressions while watching emotionally provocative silent videos, and the other partner, the ‘Face Watcher’, observed the face of the Movie Watcher. Ratings of the emotional experience (dial rotation) were acquired simultaneously for both participants at the end of each 15 s block of similar videos (each 3–4 s in duration). Both affect ratings and facial classifications were applied as modulators of face-processing neural data to highlight the neural systems that were active during live viewing of facial expressions.

The videos viewed by the Movie Watcher included ‘neutrals’, featuring landscapes, ‘adorables’, featuring animal antics, ‘creepies’, featuring spiders, worms and states of decay, intended to induce a variety of facial expressions and emotional responses that varied from positive to negative although no specific affect was targeted. The Movie Watcher rated his/her emotional valence and intensity following each 15 s video epoch. The Face Watcher rated his/her affect based on expressions on the Movie Watcher's face. The correlation of affect ratings between the Movie Watcher and Face Watcher dyads confirmed that the affect was transmitted by facial expressions, and was taken as a measure of ‘emotional contagion’. The correlation between the first principal component, PCA1, of all facial AUs of the Movie Watcher's and Face Watcher's faces was taken as a measure of facial mimicry. As such, the effects of facial mimicry and emotional contagion were both acquired simultaneously in the live interactive paradigm.

A primary aim of this investigation was to isolate the neural systems that underlie each of these dyadic functions. Specifically we test two alternative hypotheses related to neural organization. The co-occurrence of facial mimicry and emotional contagion suggests that the underlying neural systems may indeed be a common system. Alternatively, the complexity of contributing motor, social and visual functions suggests that the underlying neural systems may be separate.

In the case of facial mimicry (figure 5*a*), bilateral motor (pre- and supplementary motor cortex) and dorsolateral prefrontal cortex, DLPFC, are prominently featured and provide the face-validity of the expected motor finding. The additional components consisting of supramarginal gyrus, SMG, and superior temporal gyrus, STG, are also well-known components of the live interactive face system [32–34,96]. Together, findings of this study suggest that these neural systems support the processes of facial mimicry during the exchange of emotional information. In the case of emotional contagion during the same experimental conditions (figure 5*b*), clusters of neural activity include bilateral lateral occipital cortex, LOC, a ventral stream face-processing component, angular gyrus, AG, a social and interactive face-processing region, and the dorsal parietal region of SMG, previously observed in live face-processing tasks [34]. Together, these separate neural systems suggest that the processes of emotional contagion are distinct from those engaged during simultaneous facial mimicry. Comparison of the activity in figure 5*a*,*b* indicates that these regions are not shared between facial mimicry and emotional contagion. We note that this neural finding is also consistent with the apparent lower correlation of facial mimicry (*r* = 0.36 ± 0.11 s.e.m.) as compared with the correlation of emotional ratings (0.67 ± 0.04 s.e.m.), which further suggests that pathways for emotional contagion and physical mirroring of these emotional responses are indeed processed separately. Future investigation of functional connections and interactive properties between these neural component is suggested by these findings.

### (b) Limitations and advantages

Optical imaging of human brain function using fNIRS is limited by the shallow signal source, which is 1.5–2.0 cm from the surface. This restricts interrogation of the neural systems to superficial cortex. Thus, theoretical frameworks emerging from the haemodynamic dual-brain techniques using fNIRS represent only a subset of the working brain. However, this limitation is balanced with the advantages of imaging live social interactions that cannot be imaged by conventional magnetic resonance imaging owing to the single-person limitation. Technology provided by fNIRS is foundational for novel investigations of live social interactions. Live two-person interactive neuroscience extends the single-subject knowledge base of social behaviour and neural correlates to an emerging knowledge-based related to dyadic behavioural functions. The supporting role of multi-modal complementary approaches is also highlighted with optical imaging techniques as these approaches are not encumbered by physical constraints of the scanner or a high magnetic field. The acquisition of simultaneous behavioural information including eye-tracking, facial classification and subjective reports, for example, extends the model components that enrich neural models of live and spontaneous facial processing. However, these novel applications raise new standards for methodological validations. In particular, automated classifications of facial AUs (expressions) are currently under development. Although state-of-the-art technology has been applied here, future improvements in these methods may increase precision of these findings. A future direction for dyadic studies of emotional contagion and facial mimicry includes cross-brain neural coupling as an emerging cornerstone for interactive neuroscience. Although beyond the scope of this initial investigation, future studies using these techniques can be designed to further investigate neural coupling, functional connectivity and related neural mechanisms that underlie dyadic interactions included in emotional contagion and facial mimicry.

## Data Availability

The datasets analysed for this study are available from the Dryad Digital Repository: https://doi.org/10.5061/dryad.dz08kps16 [97] and in the electronic supplementary material [98].
